# Adaptive core-enhanced latent factor model for highly accurate QoS prediction

**DOI:** 10.3389/fdata.2026.1775728

**Published:** 2026-02-02

**Authors:** Siqi Ai, Peixin Li, Hao Fang, Yonghui Xia

**Affiliations:** 1 Beijing Mybull Technology Co., Ltd., Beijing, China; 2 College of Computer and Information Science, Southwest University, Chongqing, China

**Keywords:** adaptive regularization, latent factor (LF) model, Proportional-Integral-Derivative (PID) control, QoS prediction, quality of service (QoS), representation learning

## Abstract

Accurate prediction of Quality of Service (QoS) plays a crucial role in service recommendation and selection across large-scale distributed environments. Latent factor (LF) models have become a mainstream solution for QoS prediction owing to their simplicity and scalability, yet typical formulations struggle to capture complex latent interactions and usually rely on manually tuned regularization, which often limits prediction accuracy. To address these challenges, we propose an Adaptive Core-Enhanced Latent Factor (ACELF) model that integrates a learnable core interaction mechanism with an incremental Proportional-Integral-Derivative (PID)-driven adaptive regularization strategy. Specifically, a learnable core interaction matrix is introduced to model interactions between latent user and service factors, enabling richer representation learning beyond standard bilinear assumptions. To further enhance robustness, we design an incremental PID controller that dynamically adjusts the regularization coefficient of the core interaction matrix according to the training dynamics, allowing the optimization process to automatically balance model expressiveness and overfitting. Extensive experiments on real-world QoS datasets demonstrate that ACELF consistently outperforms several state-of-the-art methods in terms of prediction accuracy.

## 1 Introduction

With the rapid proliferation of service-oriented and cloud-based applications (Syed et al., 2025; Sadat and Dai, 2025; Boiko et al., 2024; Saad et al., 2024; Mohamed Hadjkouider et al., 2024; Jia et al., 2024), users increasingly rely on distributed service platforms to select and invoke online services. In such ecosystems, Quality of Service (QoS) information—such as response time, throughput, and availability—plays a fundamental role in determining user satisfaction and system performance (Chen et al., 2025; Yang et al., 2025; Ghafouri et al., 2022; Zhang et al., 2024; Cao et al., 2024; Wang et al., 2019). However, directly obtaining complete QoS measurements is often infeasible due to the dynamic, heterogeneous, and large-scale nature of service environments (Gnanasekaran et al., 2022; Huang et al., 2022; Jia et al., 2023). Consequently, accurate QoS prediction has become a critical research problem and has attracted significant attention in both industry and academia (Syu and Wang, 2021; Kimbugwe et al., 2021; Zheng et al., 2022).

**Figure 1:**
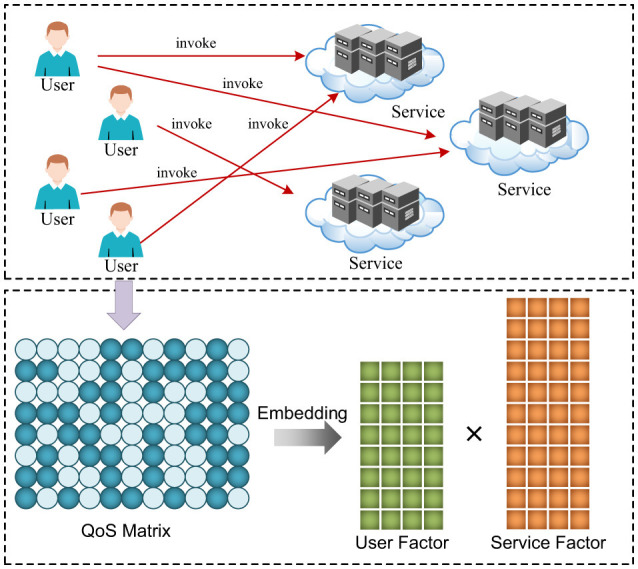
Illustration of QoS prediction using latent factor models. Given a partially observed QoS matrix, the goal is to predict missing QoS values by learning latent representations of users and services.

Among various QoS prediction techniques, latent factor (LF) models have emerged as one of the most effective and scalable paradigms (Wu et al., 2023b; Uta et al., 2024; Zhang et al., 2022; Merabet and Benmerzoug, 2022; Chen et al., 2022b, 2024b; Luo et al., 2021; Wu et al., 2023a; Chen et al., 2022a; Wu et al., 2022; Qin et al., 2024). Figure 1 illustrates the basic idea of LF models for QoS prediction. By embedding users and services into a shared low-dimensional latent space, LF models learn compact representations that enable efficient QoS estimation even under sparse observations (Jawabreh and Taweel, 2024). Wu et al. (2023b) proposed D2E-LF, an ensemble LF model that combines inner-product and distance spaces with both *L*_1_ and *L*_2_ losses. Merabet and Benmerzoug (2022) proposed an Auto-NF framework that reduces sparsity via neighbor clustering and mitigates overfitting through an autoencoder-based LF selection mechanism. Qin et al. (2024) proposed a series of adaptively accerated LF models. Chen et al. (2022a) proposed a context-aware LF model that captures low- and high-order interactions among users, services, and contextual features. Luo et al. (2021) proposed multiple extended-stochastic-gradient-based optimizers to enhance the convergence performance of LF models. Chen et al. (2024b) proposed a non-negative LF model enhanced with generalized Nesterov acceleration and particle-swarm-based hyperparameter adaptation. Xu et al. (2023) proposed an extended-linear-biases LF model with self-adaptive bias scaling. Despite their effectiveness, conventional LF formulations generally employ a simple bilinear interaction between latent vectors (Jawabreh and Taweel, 2024; Ahmadian et al., 2025). This constraint limits their ability to capture more complex, higher-order dependencies that often exist in real-world QoS data. Recent advances in graph learning have highlighted the importance of modeling complex interactions and dependencies in sparse and high-dimensional data (Bi et al., 2025a; Zhou et al., 2024; He et al., 2021, 2024; Bi et al., 2025b). For example, Bi et al. (2025a) proposed a dynamic graph mixer framework to capture coupled interactions in nonstandard tensor data. Zhou et al. (2024) developed a general representation learning framework that emphasizes structured interaction modeling. He et al. (2021, 2024) introduced advanced interaction operators for graph-based representation learning, enabling more expressive modeling of complex relational patterns. While these approaches are highly expressive, they rely on complex architectures and incur substantial computational overhead, which limits their scalability for large-scale QoS prediction.

In this work, we propose an *Adaptive Core-Enhanced Latent Factor* (ACELF) model to directly address the above challenges by jointly enriching interaction modeling and enabling dynamic regularization control. ACELF introduces a learnable core interaction matrix to modulate the interactions between latent user and service factors. This design greatly increases the flexibility of the representation space and allows the model to capture non-bilinear relationships that are impossible to express with traditional LF methods. To regulate this expressive core while preventing overfitting, we develop an incremental Proportional–Integral–Derivative (PID) controller that automatically adjusts the regularization coefficient of the core tensor during training. Unlike fixed or manually tuned regularization, the PID mechanism derives its adjustment from the optimization dynamics, providing a principled and responsive way to maintain training stability and model generalization.

The main contributions of this work can be summarized as follows:

- We propose ACELF, a novel latent factor model enhanced with a learnable and lightweight core interaction matrix to capture complex user–service interaction patterns.
- We introduce an incremental PID-based adaptive regularization strategy that dynamically tunes the core interaction matrix's regularization strength based on training feedback.
- We provide comprehensive empirical evaluations demonstrating that ACELF achieves superior accuracy and stability compared with state-of-the-art LF models.

The remainder of this paper is organized as follows. Section 2 provides preliminaries, including the LF model for QoS prediction, SGD-based learning of LF models, and the PID controller. Section 3 presents the proposed ACELF model and its optimization procedure. Section 4 reports experimental results and analysis. Section 5 concludes the paper.

## 2 Preliminaries

In this section, we briefly introduce the basic LF model for QoS prediction, its stochastic gradient descent (SGD) based learning procedure, and the PID controller. Table 1 summarizes the main notations used in this paper.

**Table 1:** Summary of main notations used in this paper.

**Symbol**	**Description**
U, S	Sets of users and services
*M*, *N*	Numbers of users and services
**R**	Partially observed QoS matrix
*r*_*us*_	Observed QoS value of user *u* on service *s*
${\hat{r}}_{us}$	Predicted QoS value of user *u* on service *s*
Ω	Index set of observed entries in **R**
Ω_tr_, Ω_val_, Ω_te_	Training, validation, and test index sets
*d*	Latent dimension
**p**_*u*_, **q**_*s*_	Latent vectors of user *u* and service *s*
**P**, **Q**	Stacked user/service latent factor matrices
**G**	Core interaction matrix modeling cross-dimension interactions
μ, *b*_*u*_, *c*_*s*_	Global mean, user bias, and service bias
L	Objective function
λ	Regularization coefficient in standard LF models
λ_*P*_, λ_*Q*_	Regularization coefficients for **P** and **Q**
λ_*G*_	Regularization coefficient for **G**
$\lambda_{G}^{\left(k\right)}$	Value of λ_*G*_ at epoch *k*
λ_min_, λ_max_	Lower and upper bounds for clipping λ_*G*_
*e*_*us*_	Per-entry prediction residual $r_{us}-{\hat{r}}_{us}$
*J*_*k*_	Validation performance indicator at epoch *k*
*J* ^*^	Reference value for PID control
*e*_*k*_	Control error at epoch *k*
*K*_*p*_, *K*_*i*_, *K*_*d*_	PID proportional, integral, and derivative gains
$\Delta \lambda_{G}^{\left(k\right)}$	Incremental PID update of λ_*G*_ at epoch *k*
η, η_*P*_, η_*Q*_, η_*G*_	Learning rates (general / for **P**, **Q**, **G**)
∥·∥_2_, ∥·∥_*F*_	Euclidean norm and Frobenius norm
〈·, ·〉_*F*_	Frobenius inner product

### 2.1 LF Model for QoS prediction

Let U={1,…,M} denote the set of users and S={1,…,N} denote the set of services. We use **R** ∈ ℝ^*M*×*N*^ to represent the partially observed QoS matrix, where *r*_*us*_ is the observed QoS value (e.g., response time or throughput) of user *u* on service *s*. The index set of observed entries is denoted by


$$\Omega =\left\{\left(u,s\right)|r_{us}\text{ is observed}\right\}.$$

LF models assume that each user *u* and each service *s* can be embedded into a shared *d*-dimensional latent space (Ahmadian et al., 2025; Lin et al., 2025b). Specifically, we associate user *u* with a latent vector ${\text{p}}_{u}\in {\mathbb{R}}^{d}$ and service *s* with a latent vector ${\text{q}}_{s}\in {\mathbb{R}}^{d}$. The predicted QoS value is then given by a bilinear interaction:


$$\begin{array}{l} {\hat{r}}_{us}={\text{p}}_{u}^{⊤}{\text{q}}_{s}, \end{array}$$ (1)

where ${\hat{r}}_{us}$ denotes the predicted QoS of user *u* on service *s*.

Let matrices $\text{P}={\left[{\text{p}}_{1},\ldots ,{\text{p}}_{M}\right]}^{⊤}\in {\mathbb{R}}^{M\times d}$ and $\text{Q}={\left[{\text{q}}_{1},\ldots ,{\text{q}}_{N}\right]}^{⊤}\in {\mathbb{R}}^{N\times d}$ collect all user and service latent factors, respectively. A common learning approach is to minimize the following regularized squared loss:


$$\begin{array}{l} L\left(\text{P},\;\text{Q}\right)=\frac{1}{2}\underset{\left(u,s\right)\in \Omega }{\sum }{\left(r_{us}-{\text{p}}_{u}^{⊤}{\text{q}}_{s}\right)}^{2}+\frac{\lambda }{2}\overset{M}{\underset{u=1}{\sum }}∥{\text{p}}_{u}{∥}_{2}^{2}+\frac{\lambda }{2}\overset{N}{\underset{s=1}{\sum }}∥{\text{q}}_{s}{∥}_{2}^{2}, \end{array}$$ (2)

where λ > 0 is a regularization coefficient that helps prevent overfitting by penalizing large latent factor norms.

### 2.2 SGD-based learning of latent factor models

SGD is a standard optimization method for large-scale machine learning problems (Wang and Joshi, 2021; Tian et al., 2023). Consider an objective function of the form


$$\begin{array}{l} L\left(\theta \right)=\underset{i}{\sum }\ell \left(\theta ;\xi_{i}\right), \end{array}$$ (3)

where ***θ*** denotes the model parameters and ℓ(***θ***; ξ_*i*_) is the loss associated with a single training sample ξ_*i*_. Instead of computing the full gradient $\nabla_{\theta }L\left(\theta \right)$ over all samples, SGD updates the parameters using one randomly sampled training instance at each iteration. The basic SGD update rule is


$$\begin{array}{l} \theta_{t+1}=\theta_{t}-\eta_{t}\nabla_{\theta }\ell \left(\theta_{t};\xi_{t}\right), \end{array}$$ (4)

where η_*t*_ > 0 is the learning rate at iteration *t*, and ξ_*t*_ is a randomly chosen sample at iteration *t*.

For the LF objective in (2), we focus on a single observed entry (*u, s*) ∈ Ω. Define the prediction error


$$\begin{array}{l} e_{us}=r_{us}-{\hat{r}}_{us}=r_{us}-{\text{p}}_{u}^{⊤}{\text{q}}_{s}. \end{array}$$ (5)

The gradients of L with respect to **p**_*u*_ and **q**_*s*_ contributed by this entry are


$$\begin{array}{l} \frac{\partial L}{\partial {\text{p}}_{u}}=-e_{us}{\text{q}}_{s}+\lambda {\text{p}}_{u}, \end{array}$$ (6)

$$\begin{array}{l} \frac{\partial L}{\partial {\text{q}}_{s}}=-e_{us}{\text{p}}_{u}+\lambda {\text{q}}_{s}. \end{array}$$ (7)

Applying the general SGD rule to (6)–(7), the parameter updates for a sampled pair (*u, s*) ∈ Ω with learning rate η > 0 become


$$\begin{array}{l} {\text{p}}_{u}\leftarrow {\text{p}}_{u}+\eta \left(e_{us}{\text{q}}_{s}-\lambda {\text{p}}_{u}\right), \end{array}$$ (8)

$$\begin{array}{l} {\text{q}}_{s}\leftarrow {\text{q}}_{s}+\eta \left(e_{us}{\text{p}}_{u}-\lambda {\text{q}}_{s}\right). \end{array}$$ (9)

By iterating these updates over multiple passes through the observed entries, the latent factors **P** and **Q** are learned in a scalable and efficient manner.

### 2.3 PID controller

The PID controller is a classical and widely used feedback control mechanism in automatic control systems (Jamil et al., 2022; Borase et al., 2021; Amertet et al., 2024). Its goal is to regulate a process variable by minimizing the deviation between a desired setpoint and the actual system output through three components: proportional (P), integral (I), and derivative (D).

Let *y*(*t*) denote the measured process variable at time *t*, and let y^*^ denote the desired setpoint. The control error is defined as


$$\begin{array}{l} e\left(t\right)=y^{*}-y\left(t\right). \end{array}$$ (10)

The continuous-time PID control law is given by


$$\begin{array}{l} u\left(t\right)=K_{p}e\left(t\right)+K_{i}\int_{0}^{t}e\left(\tau \right)\text{d}\tau +K_{d}\frac{\text{d}e\left(t\right)}{\text{d}t}, \end{array}$$ (11)

where *u*(*t*) is the control signal, and *K*_*p*_, *K*_*i*_, and *K*_*d*_ are the proportional, integral, and derivative gains, respectively. The proportional term reacts to the current error, the integral term accumulates past errors to eliminate steady-state bias, and the derivative term anticipates future trends by considering the rate of change of the error.

In discrete-time settings with sampling index *k*, the error at step *k* is denoted by *e*_*k*_. A commonly used incremental form of the discrete PID controller updates the control signal via


$$\begin{array}{l} \Delta u_{k}=u_{k}-u_{k-1}=K_{p}\left(e_{k}-e_{k-1}\right)+K_{i}e_{k}+K_{d}\left(e_{k}-2e_{k-1}+e_{k-2}\right), \end{array}$$ (12)

where *u*_*k*_ is the control signal at step *k*, and Δ*u*_*k*_ denotes its increment. The new control signal is then obtained by


$$\begin{array}{l} u_{k}=u_{k-1}+\Delta u_{k}. \end{array}$$ (13)

PID controllers have been extensively applied in various engineering domains, such as process control, robotics, and industrial automation, due to their simplicity, robustness, and ease of implementation.

## 3 Proposed method

**Figure 2:**
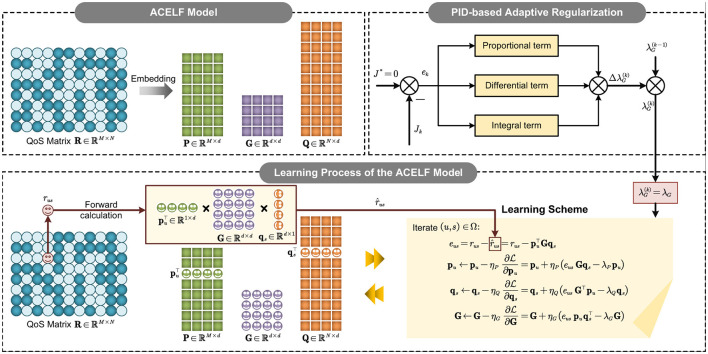
Overview of the proposed ACELF model. ACELF enhances the standard LF model by introducing a learnable core interaction matrix **G** to capture complex user–service relationships. An incremental PID controller dynamically adjusts the regularization strength λ_*G*_ of the core interaction matrix based on training feedback, enabling adaptive regularization.

In this section, we present the proposed *Adaptive Core-Enhanced Latent Factor* (ACELF) model for QoS prediction. Figure 2 illustrates the overall framework of ACELF. We first introduce the core-enhanced latent factor formulation that incorporates a learnable interaction structure between users and services, and then design an incremental PID–based strategy to adaptively tune the regularization strength of the core interaction matrix. Finally, we derive the optimization algorithm based on SGD and analyze its computational properties.

### 3.1 Core-enhanced latent factor model

Traditional LF models predict the QoS value between user *u* and service *s* by (1), which assumes that the interaction across latent dimensions is strictly one-to-one: the *i*-th dimension of **p**_*u*_ only interacts with the *i*-th dimension of **q**_*s*_ (Wu et al., 2025a). Such a restriction may be too rigid to model complex user–service relationships (Wu et al., 2024).

To enhance the expressiveness of the interaction mechanism, we introduce a learnable core interaction matrix


$$\begin{array}{l} \text{G}\in {\mathbb{R}}^{d\times d}, \end{array}$$ (14)

and define the prediction as


$$\begin{array}{l} {\hat{r}}_{us}={\text{p}}_{u}^{⊤}\text{G}{\text{q}}_{s}. \end{array}$$ (15)

Equivalently, (15) can be written in component-wise form as


$$\begin{array}{l} {\hat{r}}_{us}=\overset{d}{\underset{i=1}{\sum }}\overset{d}{\underset{j=1}{\sum }}G_{ij}p_{u,i}q_{s,j}, \end{array}$$ (16)

where *p*_*u, i*_ and *q*_*s, j*_ denote the *i*-th and *j*-th components of **p**_*u*_ and **q**_*s*_, respectively, and *G*_*ij*_ is the (*i, j*)-th entry of **G**.

From (16), we see that **G** explicitly models cross-dimension interactions: the *i*-th latent dimension of the user can interact with the *j*-th latent dimension of the service with strength *G*_*ij*_. This generalizes the standard LF model in the following sense:

- If **G** = **I**_*d*_ (identity matrix), then

$$\begin{array}{l} {\hat{r}}_{us}={\text{p}}_{u}^{⊤}{\text{I}}_{d}{\text{q}}_{s}={\text{p}}_{u}^{⊤}{\text{q}}_{s}, \end{array}$$ (17)

and ACELF reduces exactly to the conventional LF model.

- If **G** is restricted to be diagonal, i.e., **G** = diag(***γ***) with ***γ*** ∈ ℝ^*d*^, then

$$\begin{array}{l} {\hat{r}}_{us}=\overset{d}{\underset{i=1}{\sum }}\gamma_{i}p_{u,i}q_{s,i}, \end{array}$$ (18)

which corresponds to a LF model with dimension-wise reweighting of latent interactions.

- With a full matrix **G**, ACELF can capture arbitrary linear mixing between latent dimensions, substantially increasing representational capacity compared with standard LF models.

By stacking user and service latent factors into matrices


$$\begin{array}{l} \text{P}={\left[{\text{p}}_{1},\ldots ,{\text{p}}_{M}\right]}^{⊤}\in {\mathbb{R}}^{M\times d},\;\text{Q}={\left[{\text{q}}_{1},\ldots ,{\text{q}}_{N}\right]}^{⊤}\in {\mathbb{R}}^{N\times d}, \end{array}$$ (19)

the predictions for all user–service pairs can be written in a compact matrix form as


$$\begin{array}{l} \hat{\text{R}}=\text{P}\text{G}{\text{Q}}^{⊤}, \end{array}$$ (20)

where $\hat{\text{R}}\in {\mathbb{R}}^{M\times N}$ contains predicted QoS values. If we view R as a third-order tensor with modes corresponding to users, services, and an (implicit) interaction mode, then (20) can be interpreted as a degenerate Tucker-style factorization (Song et al., 2020; Wu et al., 2025c), where **P** and **Q** play the role of factor matrices and **G** serves as the core tensor along the latent dimension.

In many practical implementations, one may also include bias terms, such as a global mean μ, user bias *b*_*u*_, and service bias *c*_*s*_, leading to


$$\begin{array}{l} {\hat{r}}_{us}=\mu +b_{u}+c_{s}+{\text{p}}_{u}^{⊤}\text{G}{\text{q}}_{s}. \end{array}$$ (21)

For clarity of exposition, we focus on the interaction term and omit bias terms in the following derivations, they can be incorporated straightforwardly if needed.

**Objective Function**. Given the prediction model in (15), we define the following loss over the observed entries:


$$\begin{array}{r} L\left(\text{P},\text{Q},\text{G};\lambda \right)=\frac{1}{2}\underset{\left(u,s\right)\in \Omega }{\sum }{\left(r_{us}-{\text{p}}_{u}^{⊤}\text{G}{\text{q}}_{s}\right)}^{2}+\frac{\lambda_{P}}{2}\overset{M}{\underset{u=1}{\sum }}∥{\text{p}}_{u}{∥}_{2}^{2}\\ +\frac{\lambda_{Q}}{2}\overset{N}{\underset{s=1}{\sum }}∥{\text{q}}_{s}{∥}_{2}^{2}+\frac{\lambda_{G}}{2}∥\text{G}{∥}_{\text{2}}^{2}, \end{array}$$ (22)

where λ_*P*_, λ_*Q*_, and λ_*G*_ are nonnegative regularization coefficients. The terms $\frac{\lambda_{P}}{2}\underset{u}{\sum }∥{\text{p}}_{u}{∥}_{2}^{2}$ and $\frac{\lambda_{Q}}{2}\underset{s}{\sum }∥{\text{q}}_{s}{∥}_{2}^{2}$ constrain the magnitude of user and service latent factors, similar to standard LF models. The term $\frac{\lambda_{G}}{2}∥\text{G}{∥}_{\text{F}}^{2}$ controls the complexity of the interaction structure. A large λ_*G*_ encourages **G** toward small-norm solutions (e.g., close to the zero matrix), effectively simplifying the interaction pattern, whereas a small λ_*G*_ allows more complex cross-dimension interactions at the risk of overfitting.

The introduction of **G** brings about an intrinsic trade-off between model capacity and generalization. Compared with traditional LF (which corresponds to the special case **G** = **I**_*d*_ with no additional parameters), ACELF introduces *d*^2^ extra parameters in **G**. Properly regularizing **G** is thus crucial for avoiding overfitting, especially when *d* is moderate or large. This observation motivates the adaptive treatment of λ_*G*_, rather than fixing it manually.

**Interpretability**. Using the Frobenius inner product 〈·, ·〉_F_, the core-enhanced prediction in (16) can be rewritten as


$$\begin{array}{l} {\hat{r}}_{us}=\overset{d}{\underset{i=1}{\sum }}\overset{d}{\underset{j=1}{\sum }}G_{ij}p_{u,i}q_{s,j}={\langle \text{G},{\text{p}}_{u}{\text{q}}_{s}^{⊤}\rangle }_{\text{F}}, \end{array}$$ (23)

which indicates that the core interaction matrix **G** acts as a global interaction kernel over the outer products of user and service latent representations.

From an interpretability perspective, each entry of **G** can be viewed as an interaction weight between latent dimensions of users and services. A large positive value of *G*_*ij*_ suggests that a strong preference of a user on the *i*-th latent dimension, together with a strong attribute of a service on the *j*-th latent dimension, is likely to yield a higher QoS value. In contrast, negative values may indicate antagonistic interactions between the corresponding latent factors. Such patterns help reveal which latent dimensions cooperate or conflict in shaping QoS outcomes.

From a practical standpoint, the learned core interaction matrix provides meaningful insights for QoS analysis and service recommendation. By examining rows or columns of **G**, one can identify dominant latent dimensions and understand how different user preferences and service characteristics jointly influence QoS performance at a global level. Latent dimensions associated with consistently large interaction weights may correspond to critical service attributes or user sensitivity patterns that strongly affect QoS. Compared with standard bilinear LF models, whose interpretability is largely limited to individual latent factors, the core-enhanced formulation enables analysis of cross-dimension interactions. This allows not only identifying important latent factors but also understanding how combinations of user and service properties interact to impact QoS. Such interpretability can support QoS-aware service recommendation, system diagnosis, and service optimization, providing insights beyond pure prediction accuracy.

### 3.2 Incremental PID-based adaptive regularization

As discussed above, the regularization coefficient λ_*G*_ plays a central role in controlling the complexity of the core interaction structure. A small λ_*G*_ may lead to overfitting, while an overly large λ_*G*_ may underuse the expressive power of **G**. Fixing λ_*G*_ to a constant value chosen by grid search does not exploit the dynamic feedback available during training. A straightforward alternative is to employ heuristic or predefined regularization schedules, such as monotonically decreasing or piecewise constant rules. However, such open-loop strategies are fixed before training and cannot respond to the evolving optimization dynamics. In practice, the tendency toward overfitting or underfitting may vary across different training stages and datasets, making manually designed schedules suboptimal and sensitive to hyperparameter choices.

To address this issue, we treat λ_*G*_ as a time-varying control variable that is adjusted according to the training dynamics. Let the training process be indexed by discrete steps or epochs *k* = 1, 2, …. At each step *k*, we compute a scalar performance indicator *J*_*k*_ of the current model. A typical choice is the average validation loss over a held-out set:


$$\begin{array}{l} J_{k}=\frac{1}{\left|\Omega_{\text{val}}\right|}\underset{\left(u,s\right)\in \Omega_{\text{val}}}{\sum }\frac{1}{2}{\left(r_{us}-{\hat{r}}_{us}^{\left(k\right)}\right)}^{2}, \end{array}$$ (24)

where Ω_val_ is a disjoint validation index set, and ${\hat{r}}_{us}^{\left(k\right)}$ denotes the prediction at epoch *k*.

We set a reference value J^*^ (commonly zero) and define the control error


$$\begin{array}{l} e_{k}=J^{*}-J_{k}. \end{array}$$ (25)

We then employ the incremental PID controller introduced in the preliminaries to update the regularization coefficient λ_*G*_.

Specifically, we regard $\lambda_{G}^{\left(k\right)}$ as the control signal at step *k* and write the incremental PID update as


$$\begin{array}{l} \Delta \lambda_{G}^{\left(k\right)}=\lambda_{G}^{\left(k\right)}-\lambda_{G}^{\left(k-1\right)}=K_{p}\left(e_{k}-e_{k-1}\right)+K_{i}e_{k}+K_{d}\left(e_{k}-2e_{k-1}+e_{k-2}\right), \end{array}$$ (26)

where *K*_*p*_, *K*_*i*_, and *K*_*d*_ are the proportional, integral, and derivative gains, respectively. At the beginning of training, the historical error terms for the PID controller are initialized to zero. The new regularization coefficient is then obtained as


$$\begin{array}{l} \lambda_{G}^{\left(k\right)}=\lambda_{G}^{\left(k-1\right)}+\Delta \lambda_{G}^{\left(k\right)}. \end{array}$$ (27)

To keep $\lambda_{G}^{\left(k\right)}$ within a reasonable range and maintain numerical stability, we apply clipping:


$$\begin{array}{l} \lambda_{G}^{\left(k\right)}\leftarrow \min\left\{\max\left\{\lambda_{G}^{\left(k\right)},\lambda_{\min}\right\},\lambda_{\max}\right\}, \end{array}$$ (28)

where λ_min_ ≥ 0 and λ_max_ > λ_min_ are predefined bounds.

The three terms in (26) have complementary effects:

- The proportional term *K*_*p*_(*e*_*k*_ − *e*_*k* − 1_) reacts to the change in error and provides a direct correction proportional to the most recent deviation.
- The integral term *K*_*i*_*e*_*k*_ accumulates the error and reduces long-term bias in λ_*G*_, preventing it from staying in a region that yields persistently poor validation performance.
- The derivative term *K*_*d*_(*e*_*k*_−2*e*_*k* − 1_+*e*_*k* − 2_) captures the curvature of the error trajectory and can damp rapid oscillations in λ_*G*_, improving the stability of the training process.

By embedding this closed-loop control into the learning procedure, ACELF can automatically adapt λ_*G*_ to the current stage of optimization. When the model tends to overfit (e.g., validation loss increases), the controller can increase λ_*G*_, when the model is too rigid (e.g., validation loss decreases slowly), the controller can relax the regularization to allow more expressive interactions.

### 3.3 Optimization algorithm

**Gradient Derivation**. We now derive the SGD updates for ACELF with the objective function in (22). For a single observed entry (*u, s*) ∈ Ω, the prediction error under the core-enhanced model is


$$\begin{array}{l} e_{us}=r_{us}-{\hat{r}}_{us}=r_{us}-{\text{p}}_{u}^{⊤}\text{G}{\text{q}}_{s}. \end{array}$$ (29)

The contribution of (*u, s*) to the data-fitting term is


$$\begin{array}{l} \ell_{us}=\frac{1}{2}{\left(r_{us}-{\text{p}}_{u}^{⊤}\text{G}{\text{q}}_{s}\right)}^{2}=\frac{1}{2}e_{us}^{2}. \end{array}$$ (30)

Taking derivatives, we obtain the gradients of L w.r.t. **p**_*u*_, **q**_*s*_, and **G** as


$$\begin{array}{l} \frac{\partial L}{\partial {\text{p}}_{u}}=-e_{us}\text{G}{\text{q}}_{s}+\lambda_{P}{\text{p}}_{u}, \end{array}$$ (31)

$$\begin{array}{l} \frac{\partial L}{\partial {\text{q}}_{s}}=-e_{us}{\text{G}}^{⊤}{\text{p}}_{u}+\lambda_{Q}{\text{q}}_{s}, \end{array}$$ (32)

$$\begin{array}{l} \frac{\partial L}{\partial \text{G}}=-e_{us}{\text{p}}_{u}{\text{q}}_{s}^{⊤}+\lambda_{G}\text{G}. \end{array}$$ (33)

The first terms on the right-hand side correspond to the gradient of the squared loss, and the second terms arise from the ℓ_2_ regularization.

**SGD-based Learning Scheme**. Let η_*P*_, η_*Q*_, and η_*G*_ denote the learning rates for **p**_*u*_, **q**_*s*_, and **G**, respectively. Using the general SGD rule on (31)–(33), the parameter updates for a sampled observed entry (*u, s*) ∈ Ω are


$$\begin{array}{l} {\text{p}}_{u}\leftarrow {\text{p}}_{u}-\eta_{P}\frac{\partial L}{\partial {\text{p}}_{u}}={\text{p}}_{u}+\eta_{P}\left(e_{us}\;\text{G}{\text{q}}_{s}-\lambda_{P}{\text{p}}_{u}\right), \end{array}$$ (34)

$$\begin{array}{l} {\text{q}}_{s}\leftarrow {\text{q}}_{s}-\eta_{Q}\frac{\partial L}{\partial {\text{q}}_{s}}={\text{q}}_{s}+\eta_{Q}\left(e_{us}{\;\text{G}}^{⊤}{\text{p}}_{u}-\lambda_{Q}{\text{q}}_{s}\right), \end{array}$$ (35)

$$\begin{array}{l} \text{G}\leftarrow \text{G}-\eta_{G}\frac{\partial L}{\partial \text{G}}=\text{G}+\eta_{G}\left(e_{us}{\;\text{p}}_{u}{\text{q}}_{s}^{⊤}-\lambda_{G}\text{G}\right), \end{array}$$ (36)

where λ_*G*_ in (36) is the current value $\lambda_{G}^{\left(k\right)}$ at epoch *k* provided by the PID controller.

The complete training algorithm of ACELF is summarized in Algorithm 1. The inner loop performs SGD updates over the observed entries, while the outer loop updates the regularization coefficient λ_*G*_ using the incremental PID controller.

Algorithm 1 Training procedure of ACELF. 
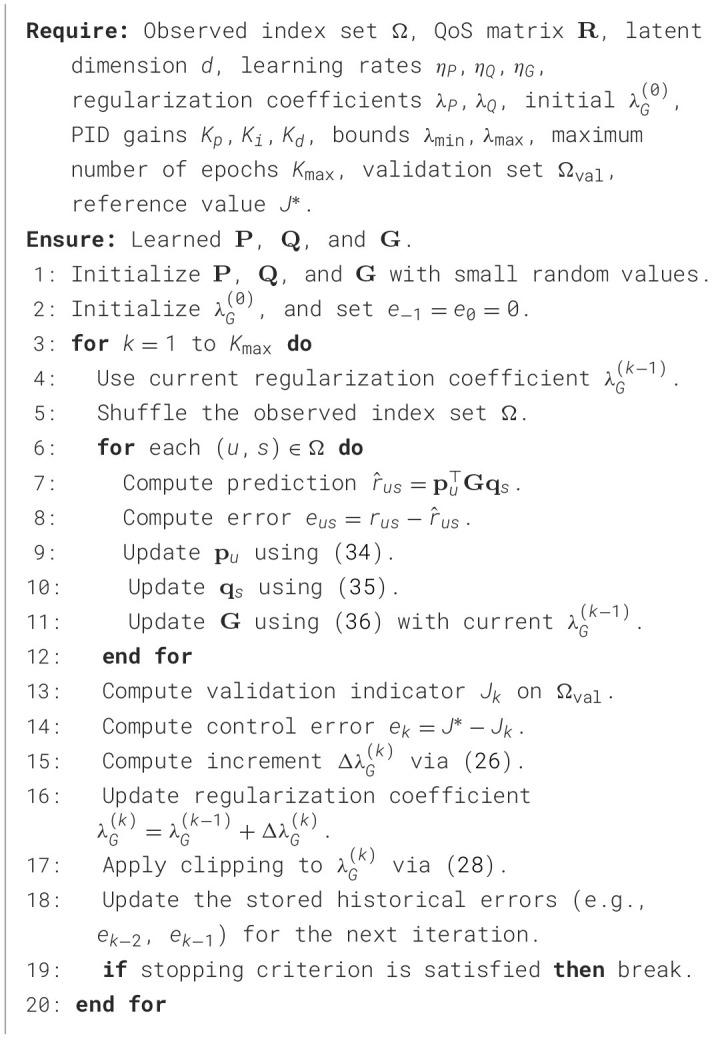

### 3.4 Computational and modeling analysis

We briefly analyze both the computational complexity and modeling characteristics of ACELF.

**Computational Complexity**. For a single observed entry (*u, s*), computing ${\hat{r}}_{us}={\text{p}}_{u}^{⊤}\text{G}{\text{q}}_{s}$ requires forming either **Gq**_*s*_ or ${\text{G}}^{⊤}{\text{p}}_{u}$, both costing *O*(*d*^2^) operations, followed by one inner product of cost *O*(*d*). The gradient computations and updates in (34)–(36) are also dominated by forming ${\text{p}}_{u}{\text{q}}_{s}^{⊤}$ and scaling **G**, which are *O*(*d*^2^). Therefore, the per-entry complexity is *O*(*d*^2^).

Let |Ω| denote the number of observed QoS entries. A full pass (epoch) over the data costs


$$\begin{array}{l} O\left(|\Omega |d^{2}\right). \end{array}$$ (37)

The additional overhead for the PID update of λ_*G*_ is *O*(1) per epoch, which is negligible compared to the SGD updates. In contrast, a standard LF model with bilinear interactions has per-entry complexity *O*(*d*), thus ACELF trades increased computational cost for stronger representational power.

**Memory Complexity**. The memory cost is dominated by storing **P** ∈ ℝ^*M*×*d*^, **Q** ∈ ℝ^*N*×*d*^, and **G** ∈ ℝ^*d*×*d*^, resulting in


$$\begin{array}{l} O\left(\left(M+N\right)d+d^{2}\right) \end{array}$$ (38)

space. When *d* is moderate, the additional *d*^2^ parameters for **G** are typically affordable in modern QoS prediction scenarios.

**Modeling Perspective**. From a modeling standpoint, ACELF strictly generalizes standard LF and provides a continuous spectrum of models controlled by λ_*G*_. When λ_*G*_ is very large, the regularization term forces **G** toward a small-norm matrix (e.g., close to the zero matrix or a scaled identity), and the model behaves similarly to a low-capacity LF model. When λ_*G*_ is very small, **G** can deviate significantly from identity and learn complex cross-dimension interactions, which increases expressiveness but may overfit the training data. By coupling ACELF with an incremental PID controller, the model can automatically adjust λ_*G*_ according to validation feedback, aiming to achieve a favorable balance between these two extremes during training.

## 4 Expreiments

In this section, we conduct empirical studies to evaluate the effectiveness of the proposed ACELF model. We first describe the experimental settings, including datasets, evaluation metrics, baselines, and implementation details. Then we report and analyze the experimental results.

### 4.1 Experimental settings

#### 4.1.1 Datasets

We evaluate ACELF on the WS-DREAM dataset, which contains QoS records collected from 339 users invoking 5,825 Web services (Lebib and Kichou, 2024). The QoS values include response time (RT) and throughput (TP), and thus the original user–service interactions can be decomposed into two matrices: RTData and TPData.

RTData contains 1,873,838 observed entries, and TPData contains 1,831,253 observed entries. To reduce the influence of heavy-tailed distributions and improve numerical stability, we apply a logarithmic scaling to the QoS values in both RTData and TPData before model training and evaluation.

To study the performance under different sparsity levels, we randomly sample observed entries from RTData and TPData at three sampling ratios (0.05, 0.075, and 0.10), forming six dataset subsets in total: **RT-0.05**, **RT-0.075**, **RT-0.10**, **TP-0.05**, **TP-0.075**, and **TP-0.10**. The statistics of these subsets are summarized in Table 2.

**Table 2:** Statistics of the QoS datasets used in our experiments.

**Dataset**	**#Users *M***	**#Services *N***	**#Observations |Ω|**	**Sparsity (%)**
RT-0.05	339	5825	93,692	95.26
RT-0.075	339	5825	140,538	92.88
RT-0.10	339	5825	187,384	90.51
TP-0.05	339	5825	91,563	95.36
TP-0.075	339	5825	137,344	93.04
TP-0.10	339	5825	183,125	90.73

#### 4.1.2 Train–validation–test split

To evaluate the generalization performance, we randomly split the observed QoS entries Ω of each subset into three disjoint sets: a training set Ω_tr_ used to learn model parameters, a validation set Ω_val_ used to tune hyperparameters and drive the PID controller, and a test set Ω_te_ used only for final performance reporting. We adopt the following split ratio:


$$|\Omega_{\text{tr}}|:|\Omega_{\text{val}}|:|\Omega_{\text{te}}|=8:1:1.$$

To reduce randomness, we repeat the random splitting process multiple times and report the average results over all runs.

#### 4.1.3 Evaluation metrics

We use standard regression metrics to assess the prediction quality of QoS values (Wu et al., 2025b; Sun and Liu, 2025; Lei et al., 2024; Lyu et al., 2026; Lin et al., 2025a; Liao et al., 2025). Given the ground-truth QoS *r*_*us*_ and the corresponding prediction ${\hat{r}}_{us}$ on the test set Ω_te_, we compute:

- **Mean Absolute Error (MAE)**:

$$\begin{array}{l} \text{MAE}=\frac{1}{\left|\Omega_{\text{te}}\right|}\underset{\left(u,s\right)\in \Omega_{\text{te}}}{\sum }\left|r_{us}-{\hat{r}}_{us}\right|. \end{array}$$ (39)

- **Root Mean Squared Error (RMSE)**:

$$\begin{array}{l} \text{RMSE}=\sqrt{\frac{1}{\left|\Omega_{\text{te}}\right|}\underset{\left(u,s\right)\in \Omega_{\text{te}}}{\sum }{\left(r_{us}-{\hat{r}}_{us}\right)}^{2}}. \end{array}$$ (40)

- **Mean Squared Error (MSE)**:

$$\begin{array}{l} \text{MSE}=\frac{1}{\left|\Omega_{\text{te}}\right|}\underset{\left(u,s\right)\in \Omega_{\text{te}}}{\sum }{\left(r_{us}-{\hat{r}}_{us}\right)}^{2}. \end{array}$$ (41)

Lower values of these metrics indicate better predictive performance.

#### 4.1.4 Implementation details

We set the latent dimension to *d* = 20 for all experiments considering the computational complexity and model capacity (Yuan et al., 2025; Chen et al., 2024a). The PID gains are empirically set to *K*_*p*_ = 0.01, *K*_*i*_ = 0.005, and *K*_*d*_ = 0.001 (Li et al., 2025).

We determine the initial learning rate and regularization strength for **G** via grid search:


$$\eta \in \left\{10^{-1},10^{-2},10^{-3}\right\},\;\lambda \in \left\{5\times 10^{-1},5\times 10^{-2},5\times 10^{-3}\right\}.$$

The best hyperparameters on TPData subsets are η = 10^−2^ and λ = 5 × 10^−2^, while on RTData subsets they are η = 10^−3^ and λ = 5 × 10^−2^. In ACELF, we apply λ to the latent factors (i.e., λ_*P*_ = λ_*Q*_ = λ), and we initialize the core regularization as $\lambda_{G}^{\left(0\right)}=\lambda$.

During training, λ_*G*_ is adaptively updated by the incremental PID controller, and we clip it to a predefined range for stability:


$$\lambda_{G}\in \left[\lambda_{\min},\lambda_{\max}\right],\;\lambda_{\min}=5\times 10^{-3},\;\lambda_{\max}=5\times 10^{-1}.$$

We run each method for at most *K*_max_ epochs. To ensure stable convergence and avoid overfitting, we adopt an early-stopping strategy based on the validation indicator *J*_*k*_ (the average validation loss). Training stops if *J*_*k*_ does not improve for *P* consecutive epochs, or if the relative improvement of *J*_*k*_ falls below a small threshold ϵ, i.e.,


$$\frac{\left|J_{k-1}-J_{k}\right|}{J_{k-1}}<\epsilon .$$

In our experiments, we set *K*_max_ = 500, *P* = 5, and ϵ = 10^−4^.

#### 4.1.5 Baselines

We compare ACELF with five representative LF-based QoS prediction baselines, covering adaptive hyperparameter tuning, PID-driven adaptation, accelerated optimization, and constrained factorization:

- **PSLF (PSO-Adaptive Latent Factor)** (Qin et al., 2024): An adaptive LF model that employs particle swarm optimization (PSO) to automatically tune key hyperparameters, including the learning rate and the regularization coefficient, during training.
- **MLF (Momentum-Accelerated Latent Factor)** (Luo et al., 2021): A momentum-enhanced LF model that accelerates convergence by incorporating classical momentum into the SGD-style updates.
- **NLF (Nesterov-Accelerated Latent Factor)** (Luo et al., 2021): An LF model optimized with Nesterov's accelerated gradient, which performs a look-ahead update along the momentum direction to achieve faster convergence.
- **PLF (PID-Adaptive Latent Factor)** (Li et al., 2025): An adaptive LF model that incorporates a PID controller to regulate the training process at the sample level, where the instantaneous per-entry prediction residual is used as the control input, and the PID-adjusted error signal replaces the original residual in the SGD updates of the standard bilinear LF model.
- **ANLF (Adaptive Non-negative Latent Factor)** (Li et al., 2023): A non-negative LF model that enforces non-negativity constraints on latent factors and adopts an adaptive learning scheme to improve robustness.

All baselines are implemented with the same latent dimension *d* = 20 and optimized using the same training protocol and early-stopping strategy as ACELF for fair comparison.

### 4.2 Comparison results and analysis

**Figure 3:**
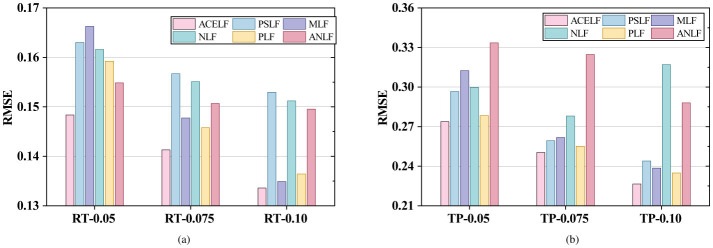
Overall RMSE comparison. **(a)** RMSE on RTData. **(b)** RMSE on TPData.

**Figure 4:**
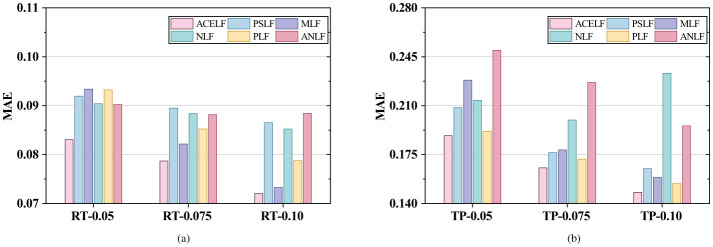
Overall MAE comparison. **(a)** MAE on RTData. **(b)** MAE on TPData.

**Figure 5:**
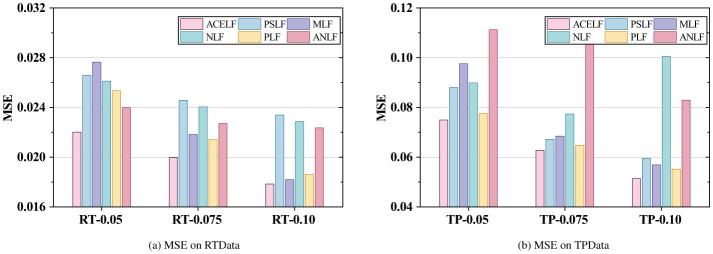
Overall MSE comparison. **(a)** MSE on RTData. **(b)** MSE on TPData.

**Figure 6:**
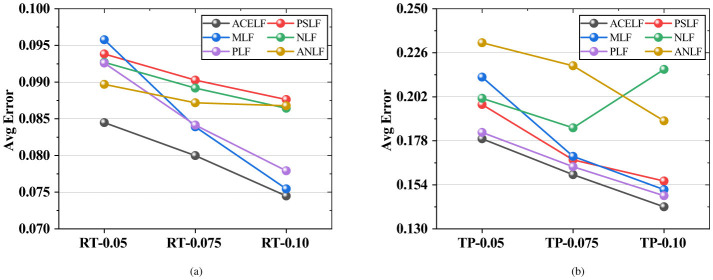
Overall Avg comparison. **(a)** Avg on RTData. **(b)** Avg on TPData.

This section presents comprehensive experimental results on six WS-DREAM subsets under different sparsity levels. Figures 3–6 visualize the RMSE, MAE, MSE, and overall Avg error results reported in Table 3, where Avg is defined as (RMSE+MAE+MSE)/3 for each dataset. Table 4 reports the per-dataset improvement of ACELF over each baseline measured by Avg error. Finally, Table 5 summarizes average performance across all datasets for each metric, and Table 6 further shows the average improvement of ACELF over baselines.

**Table 3:** Performance comparison (lower is better).

**Method**	**Metric↓**	**RT-0.05**	**RT-0.075**	**RT-0.01**	**TP-0.05**	**TP-0.075**	**TP-0.01**
ACELF	RMSE	**0.148353**	**0.141291**	**0.133584**	**0.273801**	**0.250409**	**0.226591**
	MAE	**0.083069**	**0.078701**	**0.072036**	**0.188475**	**0.165541**	**0.148113**
	MSE	**0.022009**	**0.019963**	**0.017845**	**0.074967**	**0.062705**	**0.051343**
	Avg	**0.084477**	**0.079985**	**0.074488**	**0.179081**	**0.159552**	**0.142016**
PSLF	RMSE	0.163019	0.156700	0.152960	0.296646	0.259275	0.243900
	MAE	0.091939	0.089515	0.086507	0.208619	0.176362	0.164926
	MSE	0.026575	0.024576	0.023397	0.087999	0.067223	0.059487
	Avg	0.093844	0.090264	0.087621	0.197755	0.167620	0.156104
MLF	RMSE	0.166271	0.147735	0.134903	0.312377	0.261697	0.238551
	MAE	0.093390	0.082163	0.073270	0.228292	0.178355	0.158650
	MSE	0.027646	0.021826	0.018199	0.097579	0.068486	0.056906
	Avg	0.095769	0.083908	0.075457	0.212749	0.169513	0.151369
NLF	RMSE	0.161597	0.155093	0.151201	0.299772	0.278093	0.317071
	MAE	0.090405	0.088386	0.085256	0.213895	0.199752	0.233275
	MSE	0.026114	0.024054	0.022862	0.089863	0.077336	0.100534
	Avg	0.092705	0.089178	0.086440	0.201177	0.185060	0.216960
PLF	RMSE	0.159218	0.145776	0.136415	0.278435	0.255140	0.234867
	MAE	0.093233	0.085242	0.078725	0.191721	0.171740	0.154184
	MSE	0.025350	0.021423	0.018609	0.077526	0.064752	0.055178
	Avg	0.092600	0.084147	0.077916	0.182561	0.163877	0.148076
ANLF	RMSE	0.154856	0.150690	0.149538	0.333552	0.324673	0.288066
	MAE	0.090249	0.088172	0.088414	0.249664	0.226652	0.195575
	MSE	0.023980	0.022707	0.022362	0.111257	0.105413	0.082982
	Avg	0.089695	0.087190	0.086771	0.231491	0.218913	0.188874

Avg is computed as (RMSE+MAE+MSE)/3 for each dataset. Bold values indicate the best results.

**Table 4:** Per-dataset improvement of our method over each baseline based on Avg error.

**Baseline**	**RT-0.05**	**RT-0.075**	**RT-0.1**	**TP-0.05**	**TP-0.075**	**TP-0.1**
PSLF (Δ)	0.009367	0.010279	0.013133	0.018674	0.008068	0.014089
PSLF (%)	9.98%	11.39%	14.99%	9.44%	4.81%	9.03%
MLF (Δ)	0.011292	0.003923	0.000969	0.033668	0.009961	0.009353
MLF (%)	11.79%	4.68%	1.28%	15.83%	5.88%	6.18%
NLF (Δ)	0.008228	0.009193	0.011951	0.022096	0.025509	0.074944
NLF (%)	8.88%	10.31%	13.83%	10.98%	13.78%	34.54%
PLF (Δ)	0.008123	0.004162	0.003428	0.003480	0.004326	0.006061
PLF (%)	8.77%	4.95%	4.40%	1.91%	2.64%	4.09%
ANLF (Δ)	0.005218	0.007205	0.012283	0.052410	0.059361	0.046859
ANLF (%)	5.82%	8.26%	14.16%	22.64%	27.12%	24.81%

**Table 5:** Average performance of different methods across all datasets (lower is better).

**Method**	**Avg RMSE**	**Avg MAE**	**Avg MSE**
ACELF	**0.195671**	**0.122656**	**0.041472**
PSLF	0.212083	0.136311	0.048210
MLF	0.210256	0.135687	0.048440
NLF	0.227138	0.151828	0.056794
PLF	0.201642	0.129141	0.043806
ANLF	0.233563	0.156454	0.061450

Bold values indicate the best results.

**Table 6:** Average performance improvement of our method over different baselines.

**Baseline**	**ΔRMSE**	**ΔMAE**	**ΔMSE**
PSLF	0.016412 (9.14%)	0.013655 (12.18%)	0.006738 (19.24%)
MLF	0.014584 (6.92%)	0.013031 (9.09%)	0.006968 (14.52%)
NLF	0.031466 (15.39%)	0.029172 (21.86%)	0.015322 (34.38%)
PLF	0.005970 (3.31%)	0.006485 (6.57%)	0.002334 (6.82%)
ANLF	**0.037891** (**16.93**%)	**0.033798** (**24.14**%)	**0.019978** (**37.69**%)

Bold values indicate the largest improvement over the baseline.

#### 4.2.1 Overall performance on individual metrics

From Table 3 and Figures 3–5, ACELF consistently achieves the best performance across all six subsets and all three metrics. For instance, on the most sparse RT subset (RT-0.05), ACELF achieves RMSE = 0.148353, which is lower than PSLF (0.163019), MLF (0.166271), NLF (0.161597), PLF (0.159218), and ANLF (0.154856). This trend is consistent on MAE and MSE as well: on RT-0.05, ACELF obtains MAE = 0.083069 and MSE = 0.022009, both being the lowest among all methods.

A similar conclusion holds for TP subsets. On TP-0.075, ACELF achieves RMSE = 0.250409, outperforming PSLF (0.259275), MLF (0.261697), NLF (0.278093), PLF (0.255140), and ANLF (0.324673). The margin becomes more evident when considering MSE, where ACELF obtains 0.062705 on TP-0.075, compared with 0.067223 (PSLF), 0.068486 (MLF), 0.077336 (NLF), 0.064752 (PLF), and 0.105413 (ANLF). These results show that ACELF improves both average error level (MAE) and large-error sensitivity (RMSE/MSE), indicating superior robustness.

#### 4.2.2 Overall Avg error comparison and stability across sparsity levels

The overall Avg error provides a unified view by aggregating RMSE, MAE, and MSE into one score for each dataset. As shown in Figure 6 and the “Avg” rows in Table 3, ACELF achieves the lowest Avg error on all six subsets. For example, on RT-0.075, ACELF yields Avg = 0.079985, while PSLF, MLF, NLF, PLF, and ANLF achieve 0.090264, 0.083908, 0.089178, 0.084147, and 0.087190, respectively. On TP-0.10, ACELF further reduces Avg to 0.142016, compared with 0.156104 (PSLF), 0.151369 (MLF), 0.216960 (NLF), 0.148076 (PLF), and 0.188874 (ANLF).

Importantly, ACELF shows stable superiority across different sparsity levels. On the RT side, Avg decreases monotonically as the sampling ratio increases (from 0.084477 on RT-0.05 to 0.074488 on RT-0.10), and ACELF remains the best in all cases. On the TP side, ACELF also maintains the best Avg, demonstrating that the proposed approach is robust under both sparse and relatively dense observation settings.

#### 4.2.3 Per-dataset improvements over baselines

Table 4 quantifies how much ACELF improves over each baseline on each dataset using Avg error. Several observations can be drawn.

ACELF consistently improves over all baselines on all subsets (all Δ > 0), showing that the gain is not limited to a specific competitor or a specific sparsity level. For example, compared with PSLF, ACELF reduces Avg error by 0.009367 (9.98%) on RT-0.05 and by 0.018674 (9.44%) on TP-0.05.

The relative improvements are more pronounced on TP subsets than on RT subsets, as reflected by the Avg-error reductions in Table 4. For instance, compared with ANLF, ACELF reduces the Avg error by 0.059361 (27.12%) on TP-0.07 and 0.046859 (24.81%) on TP-0.10, whereas the corresponding reduction on RT subsets is smaller (e.g., 0.005218 (5.82%) on RT-0.05). We attribute this phenomenon to the differences in data scale and error distribution between RT and TP after preprocessing, which lead to different sensitivity of the aggregated Avg metric. Nevertheless, ACELF consistently achieves the best performance on both RT and TP subsets, demonstrating the effectiveness and robustness of the proposed core-enhanced modeling and PID-driven adaptive regularization across different QoS types.

Among the compared baselines, PLF tends to be the closest competitor to ACELF on TP subsets (e.g., TP-0.05 Avg: 0.182561 vs. 0.179081), indicating that PID-based adaptation already provides some benefit. However, ACELF still achieves consistent gains, e.g., 0.003480 (1.91%) on TP-0.05 and 0.006061 (4.09%) on TP-0.10. This demonstrates that combining a more expressive core-enhanced interaction with PID-driven adaptive regularization yields additional improvements beyond controlling per-entry errors alone.

#### 4.2.4 Average performance across datasets

Table 5 summarizes the average metric values across all six datasets. ACELF achieves Avg RMSE = 0.195671, Avg MAE = 0.122656, and Avg MSE = 0.041472, which are the best among all methods. Compared with PLF, which is the second-best method in terms of average RMSE (0.201642) and average MSE (0.043806), ACELF still yields lower errors, showing that the proposed core-enhanced modeling provides consistent global benefits beyond adaptive control.

In contrast, acceleration-only baselines (MLF and NLF) are less competitive on average. For example, NLF has Avg RMSE = 0.227138 and Avg MSE = 0.056794, substantially worse than ACELF. This indicates that faster optimization alone does not guarantee better generalization in QoS prediction, while ACELF improves both optimization stability and model expressiveness.

#### 4.2.5 Average improvement over baselines

Table 6 provides an aggregated view of improvements of ACELF over baselines. ACELF consistently reduces all three metrics compared with every baseline. For example, relative to PSLF, ACELF reduces RMSE by 0.016412 (9.14%), MAE by 0.013655 (12.18%), and MSE by 0.006738 (19.24%). Relative to ANLF, the reductions are even larger: RMSE decreases by 0.037891 (16.93%), MAE by 0.033798 (24.14%), and MSE by 0.019978 (37.69%). These reductions indicate that ACELF is particularly effective at suppressing prediction errors, which is crucial in QoS prediction scenarios where occasional extreme deviations can severely affect service selection.

#### 4.2.6 Discussion

Overall, the superior results can be attributed to two complementary factors. First, the learnable core interaction in ACELF models cross-dimension dependencies between latent user and service factors, overcoming the rigid one-to-one interaction assumption of standard LF variants. Second, the PID-driven adaptive regularization dynamically adjusts the complexity of the core interaction during training, which helps prevent overfitting under sparse observations while preserving expressiveness when more complexity is needed. As evidenced by the consistent improvements across all metrics, datasets, and sparsity levels, ACELF achieves a favorable balance between representation capacity, robustness, and generalization, making it a reliable solution for QoS prediction in large-scale service environments.

## 5 Conclusions

This paper proposed ACELF, an adaptive core-enhanced latent factor model for QoS prediction. By introducing a learnable core interaction matrix, ACELF captures cross-dimension user-service interactions beyond the standard bilinear assumption, while an incremental PID mechanism adaptively adjusts the regularization strength of the core during training to balance model expressiveness and generalization. Extensive experiments on the QoS datasets under multiple sparsity settings demonstrate that ACELF consistently outperforms strong LF-based baselines in terms of RMSE, MAE, and MSE, achieving the best overall performance. Despite its effectiveness, ACELF has several limitations. First, the introduction of a full core interaction matrix increases computational cost, especially when the latent dimension grows. Second, the PID gains are empirically set and remain fixed during training, which may limit adaptability across different datasets. In future work, we plan to investigate more efficient core parameterizations, such as structured or low-rank cores, to reduce computational overhead. We also aim to explore adaptive or data-driven tuning strategies for PID parameters and to extend ACELF by incorporating richer contextual QoS information.

## Data Availability

The original contributions presented in the study are included in the article/supplementary material, further inquiries can be directed to the corresponding author.
